# Applications of artificial intelligence in cardiovascular risk detection and prediction among adults with obesity: a scoping review

**DOI:** 10.3389/fcvm.2026.1802647

**Published:** 2026-05-12

**Authors:** Mario Andrés Torres Torres, Mariana González Garcés, Jerónimo Cárdenas Montoya, Valeria Concha Fernández, Erwin Hernando Hernández Rincón

**Affiliations:** 1 Primary Care Physician, School of Medicine, Universidad de La Sabana, Chía, Colombia; 2 Primary Care Physician, Faculty of Medicine, Universidad de La Sabana, Chía, Colombia; 3 Department of Family Medicine and Public Health, Universidad de La Sabana, Chía, Colombia

**Keywords:** artificial intelligence, cardiovascular diseases, machine learning, obesity, risk assessment, risk prediction

## Abstract

**Background:**

Obesity is a chronic and multifactorial disease that substantially increases cardiovascular risk, the leading cause of mortality worldwide. Conventional cardiovascular risk prediction tools are largely derived from general populations and often fail to capture the metabolic heterogeneity and complex pathophysiology associated with obesity. Artificial intelligence (AI) has been proposed as an approach to improve cardiovascular risk detection and prediction through the integration of large and heterogeneous clinical datasets.

**Objective:**

To map and characterise the available evidence on the application of artificial intelligence for cardiovascular risk detection, prediction, and stratification in adults with obesity.

**Methods:**

scoping review was conducted following the Joanna Briggs Institute methodology and reported according to the PRISMA ScR guidelines. PubMed, Scopus, Web of Science, LILACS, and IEEE Xplore were searched for studies published between January 2015 and January 2026. Eligible studies included observational designs and investigations describing the development or validation of AI based models applied to cardiovascular risk assessment in adults with obesity. Data were synthesised using narrative and tabulated approaches.

**Results:**

Thirty studies were included, most of which were retrospective and characterised by heterogeneous populations. Tree based ensemble methods, particularly Random Forest and gradient boosting algorithms, were most frequently used, followed by support vector machines and artificial neural networks. Outcomes mainly focused on cardiovascular risk stratification and disease detection, whereas prediction of incident cardiovascular events and mortality was less common. External validation was infrequently reported, and model performance was generally moderate when longitudinal outcomes were assessed.

**Conclusions:**

Artificial intelligence shows potential as a complementary tool for cardiovascular risk assessment in adults with obesity. However, methodological heterogeneity, limited external validation, and inconsistent outcome definitions currently limit clinical implementation. Future research should prioritise prospective designs, robust external validation, and standardised outcomes to define the clinical value of AI based cardiovascular risk models in obesity.

## Introduction

Obesity is a chronic, progressive, and multifactorial disease characterised by excess adipose tissue, metabolic dysregulation, and low grade systemic inflammation. Its prevalence has increased steadily over recent decades and represents one of the most important global public health challenges. According to the World Health Organization, the global prevalence of obesity in adults has more than doubled since 1990, and in 2022 approximately 16 percent of the adult population worldwide was living with obesity (1). In absolute terms, more than one billion individuals were affected in 2022, including adults, adolescents, and children (2).

This epidemiological transition has profound health, social, and economic consequences. Obesity is associated with increased premature mortality, reduced quality of life, and greater utilisation of healthcare resources. Current projections indicate that, if prevailing trends persist, obesity prevalence will continue to rise over the coming decades, particularly in low and middle income countries (3). In this context, obesity should be understood not as an isolated clinical condition but as a major determinant of chronic non communicable diseases, most notably cardiovascular diseases.

Cardiovascular diseases remain the leading cause of mortality worldwide. In 2022, they were responsible for approximately 19.8 million deaths, accounting for nearly one third of all global deaths (4). A substantial proportion of cardiovascular morbidity and mortality is attributable to modifiable risk factors, including hypertension, dyslipidaemia, type 2 diabetes mellitus, smoking, and obesity. Consequently, accurate cardiovascular risk assessment is central to both primary and secondary prevention strategies.

International guidelines, including those issued by the European Society of Cardiology, recommend systematic cardiovascular risk stratification to guide personalised preventive interventions (5). Risk prediction tools such as SCORE2 estimate the 10 year probability of fatal and non fatal cardiovascular events using conventional clinical variables (6). However, these models were developed for general populations and present important limitations when applied to individuals with obesity, in whom cardiovascular risk is shaped by complex and interrelated metabolic, inflammatory, and haemodynamic mechanisms.

Obesity increases cardiovascular risk through multiple pathophysiological pathways, including insulin resistance, sympathetic nervous system activation, endothelial dysfunction, a prothrombotic state, and chronic inflammation, all of which contribute to atherosclerotic disease progression (7). In clinical practice, obesity frequently coexists with hypertension, dyslipidaemia, and type 2 diabetes mellitus, often clustered as metabolic syndrome, generating a cumulative and dynamic cardiometabolic risk profile. Evidence from the Global Burden of Disease study indicates that elevated body mass index contributes substantially to global cardiovascular mortality and disability (8). Beyond total adiposity, fat distribution plays a critical role, as visceral and ectopic fat depots confer excess cardiovascular risk even among individuals with similar body mass index values (9).

These features make cardiovascular risk estimation in patients with obesity particularly challenging. Conventional risk assessment tools may underestimate true risk or fail to capture obesity related heterogeneity, limiting early identification of high risk individuals and delaying the implementation of intensive preventive strategies.

In parallel with these challenges, artificial intelligence has emerged as a promising approach for cardiovascular risk modelling. Machine learning and deep learning techniques can analyse large and heterogeneous clinical datasets and capture complex non linear relationships between variables, potentially offering advantages over traditional statistical models in cardiometabolic risk assessment (10). Prior systematic and scoping reviews have described the application of artificial intelligence to cardiovascular risk prediction in broad populations, reporting variable performance, substantial methodological heterogeneity, and limited external validation (11, 12). Additional work has explored artificial intelligence based tools for obesity related vascular disease by integrating anthropometric measures, metabolic biomarkers, and lifestyle data (13).

Although obesity is commonly included as a predictor in general population cardiovascular risk models, it represents a distinct cardiometabolic phenotype characterised by complex interactions between adipose tissue distribution, inflammation, and metabolic dysfunction. These factors may influence both disease trajectories and predictive model performance. Furthermore, models developed in general populations may not adequately capture subgroup-specific risk patterns in individuals with obesity. Therefore, analysing artificial intelligence applications specifically in obese populations may provide additional insights into model behaviour and clinical relevance.

Despite this growing body of literature, important gaps remain. Existing reviews have largely focused on general populations and have not systematically mapped how artificial intelligence is applied across distinct clinical objectives in adults with obesity, including detection or diagnosis of cardiovascular disease, risk stratification, and prediction of incident cardiovascular events. Furthermore, prior syntheses have not consistently examined how model performance and clinical credibility change when moving from cross sectional or proxy outcomes to clinically meaningful incident events, or when external validation is required. As a result, the current evidence base does not clearly delineate where artificial intelligence offers potential clinical value and where important methodological and translational limitations persist in obesity focused cardiovascular risk assessment.

The aim of this scoping review is to map and characterise the available evidence on the use of artificial intelligence for detection, prediction, and stratification of cardiovascular risk in adults with obesity. By describing the types of models employed, the cardiovascular outcomes assessed, and the validation strategies reported, this review seeks to identify knowledge gaps and inform future research directions and potential clinical implementation.

## Methods

### Study design

A scoping review was conducted to systematically map and describe the available scientific evidence on the use of artificial intelligence for cardiovascular risk detection, prediction, and stratification in adults with obesity. This methodological approach was chosen to provide an overview of a heterogeneous body of literature, characterise patterns of model development and application, and identify gaps in the current evidence base, rather than to evaluate intervention effectiveness or methodological quality.

The review was conducted in accordance with the methodological guidance of the Joanna Briggs Institute for scoping reviews and is reported following the PRISMA ScR recommendations to ensure transparency and reproducibility. The study protocol was prospectively registered on the Open Science Framework and is available at https://osf.io/gw8xh. In addition to database searches, reference lists of included full text articles were manually screened to identify additional potentially relevant studies using a snowball approach.

### Research question and PCC framework

The research question was formulated using the Population, Concept, and Context framework to guide study identification and data charting.

The population comprised adults with obesity, defined according to established anthropometric criteria, including body mass index of at least 30 kg per square metre or equivalent definitions of obesity reported by the original studies, irrespective of sex or the presence of cardiometabolic comorbidities.

The concept encompassed the application of artificial intelligence methods, including machine learning, deep learning, and other algorithmic approaches, to detect, predict, or stratify cardiovascular risk or cardiovascular outcomes in adults with obesity.

The context included clinical, population based, and research settings, without geographical restriction or limitation by level of care. Eligible studies comprised observational designs and studies focused on the development or validation of predictive models that applied artificial intelligence techniques to cardiovascular risk assessment in adults with obesity.

The research question guiding this review was: What evidence is available on the use of artificial intelligence for cardiovascular risk detection, prediction, and stratification in adults with obesity?.

### Search strategy and data sources

A comprehensive literature search was conducted across five electronic databases: PubMed, LILACS, Scopus, Web of Science, and IEEE Xplore, selected for their complementary coverage of biomedical, clinical, and computational research.

Studies published between January 2015 and January 2026 were eligible for inclusion and were limited to publications in English or Spanish. This time frame was selected to capture the period during which contemporary artificial intelligence methods became increasingly applied in cardiovascular and metabolic research, in parallel with expanded availability of electronic health records, structured clinical datasets, and computational resources.

The search strategy combined controlled vocabulary terms and free text keywords related to obesity, artificial intelligence, machine learning, deep learning, cardiovascular risk, risk prediction, and risk stratification, using Boolean operators AND and OR The complete search algorithms applied across all databases are detailed in Table 1. Terms unrelated to the objectives of this review were excluded. Detailed search strategies for each database are provided in the Spplementary Material. All retrieved records were managed using the Rayyan platform to facilitate duplicate removal and screening.

**Table 1:** Search strategies.

Database	Search algorithm
IEEE Xplore	(obesity OR obese OR “body mass index” OR BMI OR “central obesity” OR “abdominal obesity”) AND (“artificial intelligence” OR “machine learning” OR “deep learning” OR “neural network**) AND (“cardiovascular risk” OR “risk prediction” OR “risk stratification” OR “risk assessment”)
	AND (model* OR predictive OR prediction OR algorithm*)
PubMed	(obesity[tiab] OR “Body Mass Index”[Mesh])
	AND (“machine learning”[tiab] OR “deep learning”[tiab]) AND (cardiovascular[tiab])
Web of Science	TS = (obesity OR obese)
	AND (“machine learning” OR “deep learning” OR “artificial intelligence”)
	AND (“cardiovascular risk” OR “risk prediction” OR “risk assessment”)
SCOPUS	TITLE-ABS-KEY(obesity OR obese)
	AND TITLE-ABS-KEY(“machine learning” OR “deep learning” OR “artificial intelligence” OR “neural network*”) AND TITLE-ABS-KEY(“cardiovascular risk” OR “risk prediction” OR “cardiovascular event*” OR “major adverse cardiovascular event*” OR MACE)
LILACS	(mh:(Obesidad) OR tw:(obesidad OR obesity OR obese)) AND ( mh:(Inteligencia Artificial) OR tw:( “inteligencia artificial” OR “artificial intelligence” OR “machine learning” OR “aprendizaje automático” OR “deep learning” OR “aprendizaje profundo” )) AND ( mh:(Evaluación de Riesgo) OR tw:( “riesgo cardiovascular” OR “cardiovascular risk” OR “predicción de riesgo” OR “risk prediction” ))

### Study selection

After removal of duplicate records, titles and abstracts were screened independently by two reviewers. Records considered potentially eligible were assessed at full text level to confirm alignment with the PCC framework.

Studies were excluded at the full text stage if they did not focus on cardiovascular risk or cardiovascular outcomes in adults with obesity, did not apply artificial intelligence based methods, or did not provide sufficient information to support mapping of methodological or clinical characteristics. The study selection process is summarised in a PRISMA ScR flow diagram.

### Eligibility criteria

Eligible studies were original research articles published between January 2015 and January 2026, including retrospective or prospective observational studies, cohort studies, and studies describing the development or validation of predictive models using real world clinical or population data.

Studies were required to apply artificial intelligence methods to detect, predict, or stratify cardiovascular risk or cardiovascular outcomes in adults with obesity, defined by body mass index of at least 30 kg per square metre or equivalent obesity definitions, such as central obesity or visceral adiposity, as reported by the original authors.

Studies were excluded if they did not involve artificial intelligence based methods, did not include adults with obesity as the target population, or did not address cardiovascular risk or outcomes. Paediatric populations and non primary research literature, including editorials, letters, individual case reports, and protocols without reported results, were excluded. In keeping with the objectives of a scoping review, no studies were excluded on the basis of methodological quality, and no formal critical appraisal was performed.

### Data extraction

Data were charted using a structured Microsoft Excel matrix developed specifically for this review. Extracted variables included country and year of publication, study design, sample size, population characteristics, clinical or population context, type of cardiovascular outcome assessed, objective of the artificial intelligence model, algorithmic approach, data sources, predictor variables, outcome definitions, reported performance metrics, validation strategies, and key methodological limitations described by the authors.

Data extraction was performed independently by two reviewers, with discrepancies resolved through discussion and consensus.

### Data synthesis

Extracted data were summarised in tables to describe the methodological and clinical characteristics of the included studies. A narrative synthesis was conducted to map patterns in the application of artificial intelligence for cardiovascular risk detection, prediction, and stratification in adults with obesity.

Studies were grouped according to the category of cardiovascular outcome assessed, the type of artificial intelligence method applied, and the validation approach reported. The synthesis focused on describing the scope of existing evidence, methodological trends, and recurring limitations, particularly those related to outcome definition and external validation, without attempting quantitative comparison or effectiveness assessment.

## Results

**Figure 1:**
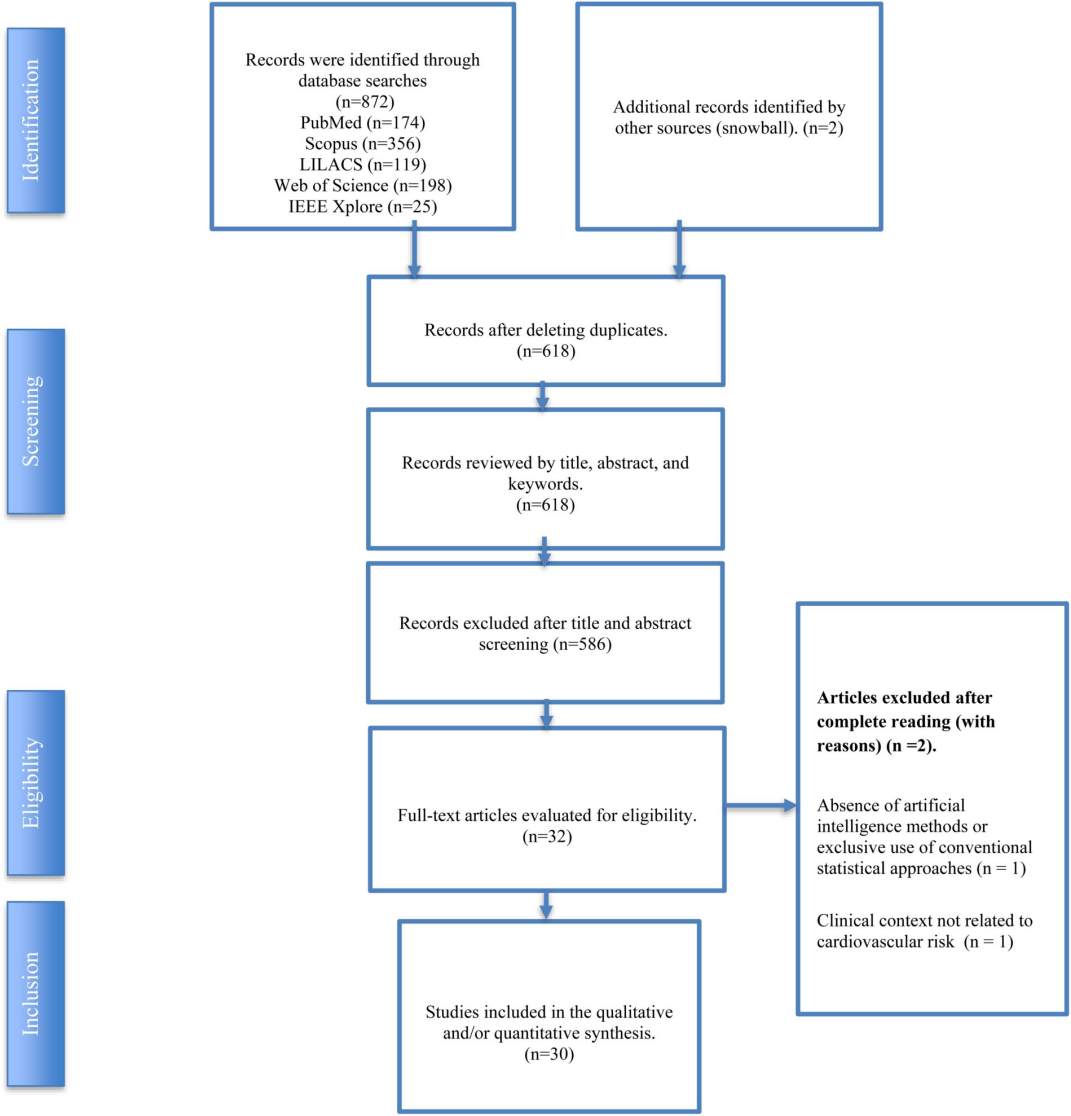
Prisma ScR.

The systematic search identified 30 studies addressing the application of artificial intelligence to cardiovascular risk assessment in adults with obesity. Twenty eight studies were retrieved through database searches and two additional studies were identified through snowball screening of reference lists. The study selection process is summarised in the PRISMA ScR flow diagram (Figure 1). In accordance with the exploratory nature of a scoping review, no studies were excluded on the basis of methodological quality.

At a global level, the included evidence exhibited consistent structural patterns. Most studies employed retrospective observational designs and focused primarily on cardiovascular risk stratification or diagnosis rather than on prospective prediction of incident cardiovascular events. Supervised machine learning models applied to structured clinical data predominated across studies, while external validation using independent populations was infrequently reported.

**Figure 2:**
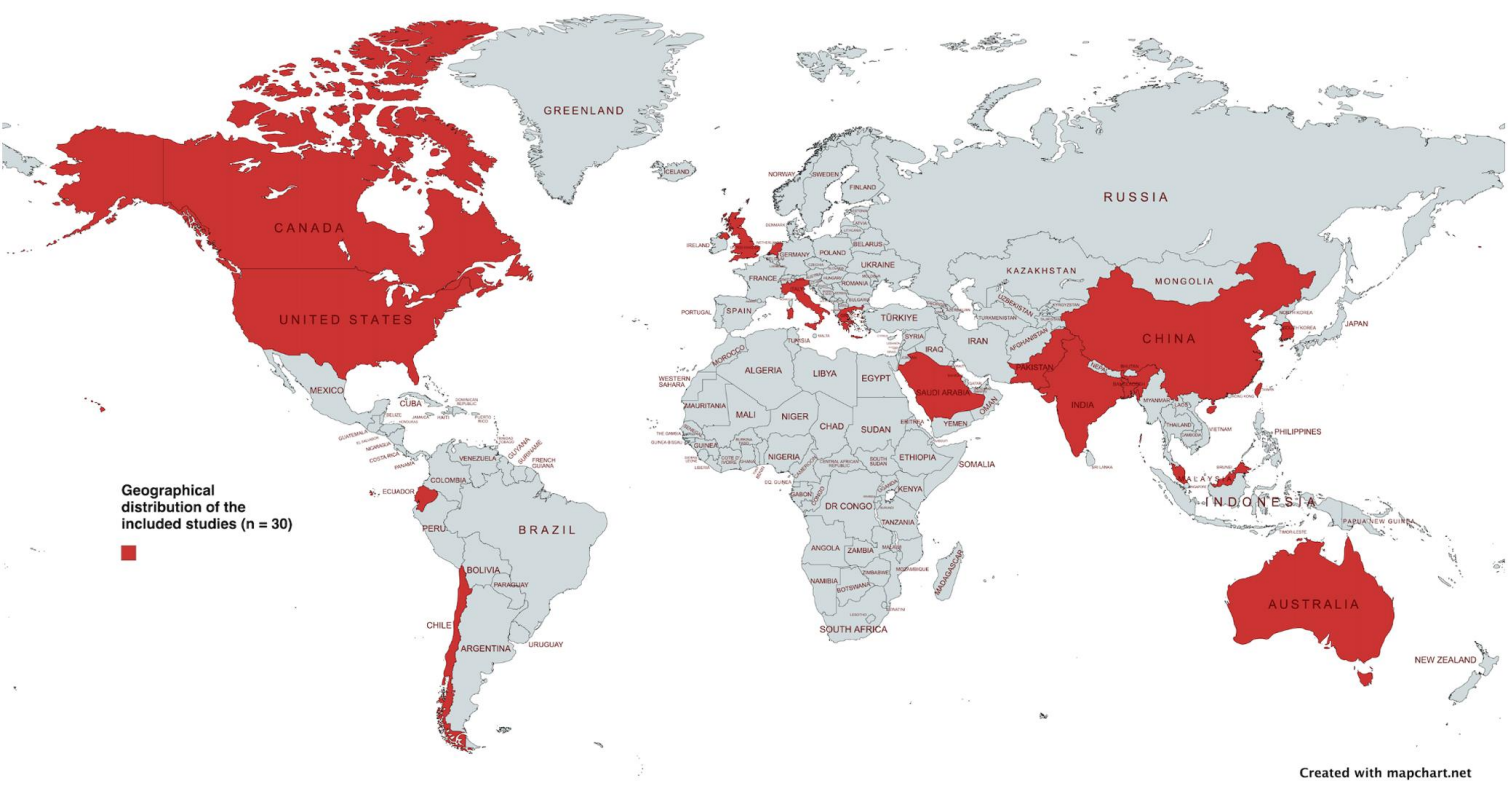
Geographical distribution of the included studies (*n* = 30). The map illustrates the geographical distribution of the included studies (*n* = 30), showing a concentration of evidence from regions with established research and data infrastructure.

The included studies were published between 2015 and 2026, with a marked increase in publication volume after 2020, reflecting growing interest in artificial intelligence based cardiovascular risk assessment in populations with obesity. Studies were conducted across diverse geographic regions, including Europe, Asia, North America, Latin America, the Middle East, and Africa, and were set in clinical, population based, occupational, and academic contexts. The geographical distribution of the included studies is shown in Figure 2, highlighting a higher concentration of evidence from regions with established data and technological infrastructure. Sample sizes varied substantially, ranging from fewer than 1,000 participants to large population-based datasets comprising tens or hundreds of thousands of individuals.

Across studies, obesity was most commonly defined using body mass index thresholds of at least 30 kg per square metre, in accordance with international criteria. Several investigations incorporated alternative or complementary adiposity measures, including central obesity, visceral adiposity, or derived anthropometric indices, aiming to better capture obesity related cardiovascular risk heterogeneity (14, 15). Study populations included adults of both sexes with variable age distributions and heterogeneous cardiometabolic profiles, including hypertension, type 2 diabetes mellitus, and dyslipidaemia. Some studies focused on specific subgroups, such as university students or occupational cohorts, whereas others analysed general population or ageing cohorts (10, 16).

All included studies applied supervised machine learning techniques. Ensemble based methods, particularly Random Forest, Extreme Gradient Boosting, and Gradient Boosting Machines, were the most frequently used algorithms, followed by Support Vector Machines and artificial neural networks. Conventional statistical approaches, including logistic regression and Cox proportional hazards models, were often employed as reference comparators alongside artificial intelligence based models (17–19). Deep learning approaches were less frequently used and were largely confined to studies with large sample sizes or multiple data sources.

Data sources included electronic health records, population based cohorts, occupational health databases, and institution specific datasets. Predictor variables were largely consistent across studies and predominantly included demographic factors such as age and sex, anthropometric measures including body mass index and waist circumference, clinical variables such as blood pressure and medical history, and biochemical markers including glucose and lipid parameters. A minority of studies incorporated lifestyle variables, composite adiposity indices, imaging data, or advanced biomarkers.

The cardiovascular outcomes modelled across studies were heterogeneous but could be coherently classified into four categories. The most frequently assessed outcomes were cardiovascular or cardiometabolic risk stratification and diagnosis of established cardiovascular disease, typically evaluated using cross sectional or retrospective designs (16, 20–24). A smaller subset of studies focused on incident cardiovascular events, including ischaemic heart disease, stroke, heart failure, or composite cardiovascular outcomes, generally using longitudinal designs and time to event analyses (17, 25, 26). Mortality outcomes, either cardiovascular or all cause, were evaluated in a limited number of large population based cohorts (27). Distinct application domains and consistent predictive performance patterns of artificial intelligence models across these outcome categories are summarised in Table 2.

**Table 2:** Artificial intelligence application domains and predictive performance patterns in cardiovascular risk assessment among adults with obesity.

AI application domain	Primary predicted outcome	Consistency of predictive performance	Key performance pattern	References
Cardiovascular risk stratification	High vs. low cardiometabolic risk	High	Ensemble ML models consistently outperformed traditional risk scores in cross-sectional stratification tasks	(15, 16, 22, 24, 28)
Diagnosis of established cardiovascular disease	Presence of CVD or cardiometabolic disease	High	Models trained on structured clinical and metabolic data showed high discrimination, particularly in retrospective settings	(14, 17, 20, 24, 29)
Incident cardiovascular event prediction	First cardiovascular event	Moderate	Predictive performance declined when longitudinal outcomes and external validation were required	(17, 25, 26, 30)
Mortality prediction	Cardiovascular or all-cause mortality	Moderate	Models incorporating disease severity hierarchies achieved clinically plausible but moderate discrimination	(27)
Obesity-related vascular disease detection	Subclinical or early vascular disease	Moderate–high	Integration of anthropometric and metabolic features enabled early vascular risk detection	(13)
Multimorbidity clustering	Cardiometabolic and mental health clusters	Exploratory	Latent class and ML approaches identified distinct risk phenotypes with limited external validation	(25)

Model performance was most commonly reported using area under the receiver operating characteristic curve, concordance index, sensitivity, specificity, and overall accuracy. Performance estimates varied widely across studies and outcome categories. Internal validation strategies, such as cross validation or training and test splits, predominated, whereas external validation across independent datasets was reported in only a minority of studies, particularly among those modelling incident cardiovascular events. The outcome domains successfully predicted by artificial intelligence models and their associated clinical implications are summarised in Table 3.

**Table 3:** Outcome domains successfully predicted by artificial intelligence models and their clinical implications in adults with obesity.

Outcome domain	Direction of prediction	Strength of evidence	Clinical implication	References
Cardiovascular risk classification	High vs. low risk	Strong	Enables population-level screening and prioritisation for preventive interventions	(15, 16, 22, 24, 28)
Diagnosis of cardiovascular disease	Disease vs. no disease	Strong	Supports early identification of established cardiovascular disease in high-risk obese populations	(14, 17, 20, 29)
Incident cardiovascular events	Event vs. no event	Moderate	Limited utility for individual prediction; more suitable for research and risk modelling	(17, 25, 26, 30)
Mortality	Increased vs. reduced risk	Moderate	May assist in long-term prognostic stratification rather than acute decision making	(27)
Obesity phenotype differentiation	Heterogeneous cardiometabolic profiles	Emerging	Highlights limitations of BMI-only definitions and supports phenotypic risk assessment	(17, 30, 31)
Decision support and risk prioritisation	Appropriate vs. delayed action	Exploratory	Potential role in clinical decision support systems pending external validation	(18, 32)

## Discussion

### Cardiovascular risk assessment in obesity and the potential role of artificial intelligence

Obesity represents a distinctive challenge for cardiovascular prevention due to the complex interplay between metabolic dysfunction, chronic inflammation, altered body fat distribution and hemodynamic changes, factors that are incompletely captured by traditional cardiovascular risk scores. In this context, artificial intelligence has emerged as a promising approach to integrate multidimensional clinical data and potentially improve early risk identification in individuals with obesity. This scoping review, which included 30 studies, provides a structured overview of how artificial intelligence has been applied to cardiovascular risk detection in this population and highlights recurring methodological patterns, strengths and limitations within the current body of evidence (13, 16, 35, 36).

### Conceptual and methodological heterogeneity of the available evidence

A central finding of this review is the substantial heterogeneity observed across included studies, both in methodological design and in the clinical objectives pursued by the proposed models. While some investigations focused on the prediction of incident cardiovascular events using longitudinal designs, others addressed cross sectional diagnosis of established cardiovascular disease or cardiometabolic risk stratification based on proxy outcomes (15, 22).

This conceptual variability limits direct comparison across models and contributes to the wide range of performance metrics reported. More importantly, it reflects an unresolved lack of consensus regarding which cardiovascular outcomes should be prioritized when developing and evaluating artificial intelligence based tools in populations with obesity.

### Outcome specific performance and clinical relevance

Model performance differed markedly according to the type of cardiovascular outcome assessed. Studies targeting incident cardiovascular events, particularly those incorporating longitudinal follow up and external validation, consistently reported moderate discrimination, with area under the curve or concordance index values rarely exceeding 0.70 (15, 26).

In contrast, cross sectional or retrospective studies evaluating prevalent disease or contemporaneous diagnoses frequently reported very high discrimination metrics, in some cases exceeding 0.90 (15, 24). From a clinical perspective, this discrepancy should not be interpreted as inferior performance of longitudinal models, but rather as a more realistic reflection of the inherent difficulty of prospective cardiovascular risk prediction in individuals with obesity.

### Algorithmic approaches and comparison with traditional models

From an algorithmic standpoint, tree based ensemble methods such as Random Forest and Extreme Gradient Boosting clearly dominated the literature (15, 17, 22, 24). This predominance is consistent with the tabular structure of most clinical datasets and the need to model complex nonlinear interactions among demographic, anthropometric and metabolic variables.

Although several studies reported superior discrimination compared with logistic regression or Cox proportional hazards models, few formally assessed incremental clinical value through calibration analyses or decision curve analysis (18, 32). Consequently, reported improvements in discrimination alone may not translate into meaningful clinical benefit.

Deep learning approaches were less frequently applied and did not demonstrate consistent superiority over ensemble methods. High performance metrics associated with neural networks were largely confined to internally validated studies with cross sectional outcomes, raising concerns regarding overfitting and limited generalizability (17, 24).

### Validation strategies and generalizability of models

Model validation emerged as a critical methodological limitation across the reviewed studies. Most investigations relied exclusively on internal validation strategies, such as random data splitting or cross validation, which are known to overestimate model performance when applied to external populations.

Only a limited number of studies incorporated external or multi cohort validation, and these consistently reported more modest yet clinically plausible performance estimates (26, 27, 33). This pattern underscores a substantial gap between methodological development and real world applicability of artificial intelligence based cardiovascular risk models in obesity.

### Methodological limitations and sources of bias in AI-based prediction models

A critical appraisal of the included studies reveals several recurrent sources of bias intrinsic to artificial intelligence based prediction research. Data leakage, particularly when preprocessing steps are performed prior to dataset splitting, was rarely addressed and may have led to overly optimistic performance estimates (10, 18). In addition, most studies lacked temporal validation, limiting the ability of models to capture real world temporal dynamics and reducing their robustness in prospective settings (10).

Another important limitation relates to the frequent use of proxy outcomes, such as composite cardiometabolic risk scores or cross sectional disease status, instead of clinically adjudicated incident cardiovascular events. This approach may inflate discrimination metrics while limiting clinical interpretability (11, 22).

Small sample sizes in several studies, especially those using complex models such as neural networks, further increase the risk of overfitting. These methodological issues, combined with inconsistent reporting of model calibration and clinical utility, highlight a substantial gap between model development and clinical applicability (18, 32).

### Definition of obesity and cardiovascular risk characterization

Another important limitation relates to how obesity was defined and operationalized within predictive models. The majority of studies relied solely on body mass index, despite growing evidence that visceral adiposity, fat distribution and metabolic phenotypes confer differential cardiovascular risk (17, 30, 31).

The limited incorporation of alternative adiposity measures constrains the ability of current models to capture clinical heterogeneity within obese populations and may lead to risk misclassification in specific subgroups, including individuals with metabolically unhealthy obesity or those with elevated body mass index but relatively favorable cardiometabolic profiles.

Beyond variability in definitions, few studies systematically evaluated whether the inclusion of more detailed adiposity measures translated into improved predictive performance. While body mass index was the most frequently used metric, alternative measures, such as waist-to-hip ratio, visceral fat indices, and body composition-based approaches, were less commonly incorporated (17, 30, 31).

Studies incorporating more refined adiposity metrics suggested potential improvements in predictive performance; however, these findings were not consistent across all studies and were often limited by small sample sizes or lack of external validation (17, 30). This inconsistency highlights the absence of standardized approaches to obesity characterization in predictive modeling.

Overall, the predominant reliance on body mass index reflects both data availability and methodological simplicity, but may limit the ability of artificial intelligence models to capture the full spectrum of cardiometabolic heterogeneity in obese populations.

### Conceptual framework for AI-based cardiovascular risk assessment in obesity

To enhance interpretability and integration of the available evidence, we propose a conceptual framework that organises artificial intelligence applications according to three key dimensions: clinical purpose, data type, and stage of model development.

From a clinical perspective, models can be broadly classified as diagnostic, focusing on identifying existing cardiovascular disease, or prognostic, aiming to predict incident cardiovascular events or mortality. This distinction is essential, as models targeting cross-sectional outcomes tend to report higher discrimination but offer limited clinical utility compared to those predicting longitudinal outcomes (11, 22, 37, 38).

In terms of data inputs, most models rely on structured clinical and biochemical variables, while fewer incorporate more complex data, such as imaging, composite adiposity indices, or multimodal datasets (17, 34, 39). This variability influences both model performance and interpretability.

Finally, the maturity of models varies substantially, ranging from early development and internal validation to limited external validation, with very few reaching implementation stages. This gap reflects broader challenges in artificial intelligence research, including the need for standardized reporting and translational applicability (18, 32, 40).

This framework may facilitate comparison across studies and help identify priorities for future research and clinical translation.

### Clinical implications and future research directions

Taken together, the current evidence does not support replacing established cardiovascular risk assessment tools with artificial intelligence-based models in adults with obesity in routine clinical practice (10, 18, 22). However, these models may have a complementary role in selected contexts, such as population level screening, risk prioritization or identification of individuals who may benefit from more intensive cardiovascular evaluation (41).

Future research should prioritize standardized, clinically meaningful outcomes; rigorous external and temporal validation; systematic reporting of calibration and clinical utility; and more precise characterization of obesity-related phenotypes. Without these advances, the promise of artificial intelligence in cardiovascular prevention among individuals with obesity is likely to remain largely exploratory (42).

Despite the growing number of studies, the current body of evidence remains methodologically immature and insufficient to support widespread clinical implementation of artificial intelligence models in cardiovascular risk assessment among individuals with obesity. Many models are developed under conditions that favor internal validity but do not reflect real-world clinical complexity, limiting their translational potential (10, 18).

Moreover, improvements in discrimination metrics alone are insufficient to justify clinical adoption. The lack of consistent evaluation of calibration, clinical utility, and decision impact raises concerns about the real benefit of these models in practice (18, 32), without rigorous external validation and alignment with clinically meaningful outcomes. Artificial intelligence applications in this field risk remaining primarily exploratory rather than transformative.

## Conclusions

The current evidence indicates that artificial intelligence based models for cardiovascular risk assessment in adults with obesity are characterized by substantial methodological heterogeneity, a predominant reliance on internal validation, limited reporting of calibration and clinical utility, and inconsistent evaluation of clinically meaningful outcomes.

When incident cardiovascular events and external or multicohort validation are required, model performance consistently declines to moderate levels. This finding does not represent a methodological failure of artificial intelligence, but rather reflects the inherent complexity of predicting prospective cardiovascular risk in populations with obesity.

At present, artificial intelligence should be regarded as a complementary and exploratory tool for cardiovascular risk detection and prioritization, rather than an autonomous decisión-making instrument in clinical practice. Meaningful progress in this field will depend less on increasing algorithmic complexity and more on clearly defined clinical questions, standardized outcome definitions, rigorous external validation, and improved characterization of obesity-related cardiovascular risk. Without these elements, the promise of artificial intelligence in cardiovascular prevention is likely to remain aspirational rather than transformative.

## Data Availability

The original contributions presented in the study are included in the article/supplementary material, further inquiries can be directed to the corresponding author/s.
